# Supplementary material for: Sexual and reproductive health research capacity strengthening programs in low- and middle-income countries: A scoping review
Source: PLOS Glob Public Health. 2024 Oct 3;4(10):e0003789. doi: 10.1371/journal.pgph.0003789 (PMC11449353; doi:10.1371/journal.pgph.0003789)
Supplement: S1 Appendix — (DOCX) [file pgph.0003789.s002.docx]

**Appendix A:** Populated data extraction form that was used to gather information for the 18 articles included in this scoping review of reproductive health research strengthening programs in low- and middle-income countries

| Lead author, title, year of publication | Country | Goal of RHR capacity strengthening program | Sample size, population | Study design | RHR intervention frequency and format | Learning activities | Reported outcomes | Key findings | Lessons learned | Sustainability | Limitations identified by researchers |
| --- | --- | --- | --- | --- | --- | --- | --- | --- | --- | --- | --- |
| Agyepong, Strengthening capacities and resource allocation for co-production of health research in low and middle income countries, 2021 | Burkina Faso,  Côte d’Ivoire, Ghana, Niger, Senegal,  and Sierra Leone. | propose a framework to structure, design, and implement  co-production capacity strengthening at  inter-related individual, institutional, and contextual levels | a mix of national and  sub-national level health professionals,  academic researchers, civil society organi-  sations, and media practitioners | case study | design and co-produce  research, interventions, and advocacy to support  improvements in women, newborn,  children, and adolescent wellbeing in six  countries in west Africa | Peer-to-peer as well as peer-to-facilitator  engagement and learning within and  across teams are encouraged using a  series of interactive workshops about conducting research including theories of knowledge, design,  conduct, synthesis, interpretation, and use. | Proposed framework on co-production capacity strengthening health research.  co-production has the potential to make  a difference to health outcomes in LMICs. | Strengthening co-production capacity requires creating space for and  allowing a diversity of knowledge  and expertise and paying attention to power dynamics between team members | Adequate human and financial  resources are needed to strengthen  capacity for the co-production of  health research | Capacity building also requires  medium to long term rather than  short term efforts to establish and  institutionalize a culture of health  research co-production | Unspecified |
| Baron, Collateral benefits: how the practical application of Good  Participatory Practice can strengthen HIV research in  sub-Saharan Africa, 2018 | SSA, unspecified number of countries | Examine Wits Reproductive Health and HIV Institute (Wits RHI) lessons  learned from implementing Good Participatory Practice (GPP), its ongoing efforts to institutionalize GPP, and the yet to be realized potential in creating fully  sustainable structures for meaningful stakeholder engagement in HIV prevention research, implementation science and  beyond. | Unspecified | Case study | Working in partnership with AIDS Vaccine Advocacy Coalition (AVAC), the Follow-on African Consortium for Tenofovir Studies (FACTS) Coordinating Team formally incorporated GPP from the early plan-  ning phase of the trial. A GPP section in the study-wide  Manual of Procedures (MOP) outlined the strategy, tactics  and support that would be employed, and a series of novel  tools were developed to support stakeholder engagement  throughout the trial. | Staff and community advisory board  (CAB) trainings were convened before activation and during the study. A GPP site preparation checklist as well as structured templates for site-specific plans were developed to address stakeholder education, engagement, communications and issues management, which were  regularly reviewed. | Obtaining upfront funding to support GPP activities throughout and between the research life cycle, and a trained multi-disciplinary team of GPP practitioners helped facilitate an enabling environment  for GPP implementation. Recommend formally integrating stakeholder engagement into study documents, including  M&E plans with indicators and performance metrics, to assist teams to track and refine their GPP strate-  gies. | Institutionalizing resources and supporting organization-wide GPP along with ongoing support can help build efficiencies and maximize economies of scale toward a pragmatic and innovative application of the GPP Guidelines. Realizing the full potential of GPP, including direct and indirect – ‘collateral benefits’  will require the collective buy-in and support from sponsors, implementers and community stakeholders across the research  field. | Need early commitment to include GPP-dedicated human resources, activities and over-  sight mechanisms into the initial grant and budget proposal. Develop social media campaigns beyond the scope of a specific trial to support relevant broad-based initiatives. Build a  research-informed and empowered network of stakeholders  that can effectively engage without research teams having to  “start from scratch” every time a new study enters the  research field. | Develop generic GPP tool, templates and SOPs than can be adopted across research projects, establish knowledge management systems for GPP resource sharing to instill institutional memory | Not all aspects  of the GPP Guidelines could be applied, due in part to high  staff turn-over. This, in turn, resulted in the need or repeat  training in GPP planning, execution and reporting. A further  challenge emerged in finding ways to formally monitor the  impact of the GPP activities without making reporting too burdensome |
| Becerril-Montekio, Using systematized tacit knowledge to  prioritize implementation challenges in existing  maternal health programs: implications for the post MDG era, 2016 | Mexico, Nicaragua | Assess the feasibility of using the  tacit knowledge methodology to prioritize challenges to implementation of current maternal health programs and inform the post  MDG agenda in LMICs. | 6 communities of practice (CoPs) formed in 3 states of Mexico and 3 departments of Nicaragua. | Quantitative, implementation | Participants attended several workshops and developed different online activities aiming to strengthen their capacities to acquire, analyze, adapt and apply research results and to systematize their experience and knowledge of  the actual implementation of these programs from July to Dec 2013. | Concept mapping, a general method designed to organize and depict the ideas of a group on a particular topic, was used to manage, discuss and systematize their tacit knowledge about implementation problems of the programs they work in. | 200 & 231 participants from 3 communities of practice in each country registered for the online concept mapping platform, 200 people completed sorting  and rating activities. Deficiencies  in the quality of maternal health care emerged as a priority issue  based on the systemized tacit knowledge of participating healthcare  personnel in both Mexico and Nicaragua. | Great similarity between the  Mexican and Nicaraguan general results highlighting the importance and the feasibility of solution of implementation problems related to the quality of healthcare. | The use of rigorously organized tacit knowledge of health personnel proved to be a feasible and useful tool for prioritization to  inform implementation priorities in the post MDG agenda. | Strategic planning should aim to work on building the necessary capacities of the health system and  the health personnel in care provision and its management. | Unspecified |
| Brizuela,,Supporting early-career women researchers: lessons from a global mentorship programme, 2023 | LMICs | Describe the process of designing and  implementing a mentorship programme for early career women SRHR researchers from LMICs including valuable  lessons learned vis-à-vis existing evidence. | 13 mentor-mentee  pairs took part, early career  women researchers | case study | completed online with remote support sessions and mentorship  meetings. Research topics included how to manage the research team in pursuit of common goals. | During the mentor training session, participants  were provided with tools and resources that they  could be used with mentees. Mentees’ train-  ing focused on how to optimize their experiences | A structured mentorship programme can offer the  necessary environment for development of mentors  and mentees alike, and for providing the basis for  a lasting relationship. | Providing women researchers with the tools through mentorship to navigate the unique challenges they face in their career journeys, can have a lasting impact on research capacity. | Participants: men were invited to apply for the  role of mentor whereas positions for mentees  remained dedicated to individuals identifying  as women. | Countries  and institutions committed to strengthening research capacity need to focus on the holistic  growth and motivation of individuals in a way that ensures gender equality. | limited by the number of  mentor applicants. Limited internet connectivity. |
| Compaoré, ‘We always find things to learn from.’  Lessons from the implementation of the  global maternal sepsis study on research  capacity: a qualitative study, 2021 | Kenya, Malawi, and Zimbabwe, Benin, Mali, and Senegal, Guatemala, Honduras, and Nicaragua | Evaluate the extent to which participation  in a multi-country maternal health study was able  to strengthen individual and institutional research capacity | Members of local research  teams that took part in Global Maternal Sepsis Study (GLOSS).  70 participants representing 16 LMICs | Qualitative study, using IDIs and FGDs | The participants in this study had previously been part of research teams in GLOSS | Participated in health research in various capacities: PI, project manager, site coordinator, data entry & management, Data Collector, Data clerk | Six themes emerged from the data analysis: recognized need for research capacity, unintended effects of  participating in research, perceived ownership and linkage with the research study, being just data collectors,  belonging to an institution that supports and fosters research, and presenting study results back to study  implementers. | Research capacity strengthening of local researchers was unintentional  outcome of the large multi-country study on maternal sepsis. | Researchers in LMICs  need continuous and long-term support to conduct  studies based on national needs and priorities. They also  need the training and exposure to research needed to  improve quality. | For sustainable research capacity to be built,  study coordinators and funders need to deliberately plan for it, addressing needs at both the individual and  institutional level.  unclear if these collaborations would  survive beyond the GLOSS project | Interviews were conducted at the participants’ workplace,  these were sometimes interrupted, potentially  impacting the openness of the participants and therefore,  the quality, one of the researchers in this  study played a role in coordinating GLOSS and this involvement  could have led to potential response bias |
| Dossou, Switching the poles in sexual and reproductive health research: implementing a research capacity-strengthening network in West and North Africa, 2016 | Benin, Burkina  Faso, Guinea, Ivory coast, Senegal, Algeria,  Morocco, Tunisia | The NetSRH aims at developing and strengthening scientific  capacities and collaborations between institutions  that conduct research in SRH that can be translated into  policy and practice. | 11 institutions. Focus on francophone countries aimed at contributing to the reduction  of the French-English gap in terms of research  capacity within the continent. | Case study: network governance model with a multi-centric  system of interaction between interdependent actors, negotiation  rationality for decision making, and insurance of  compliance through trust and political obligation | Workshops, launched a blog as a tool to promote an internal communication, Members wrote 17 research proposals, based  on research questions they perceived as locally relevant | 14 proposals underwent several rounds  of peer review, online and face-to-face | 11  Research projects directly or indirectly initiated via the network are being conducted | critical local contextual  evidence shared with ITM, about contraceptive  seeking behaviors in Benin, in a collective effort  to design a research proposal submitted to the Gate Foundation in March 2016 | Achieving the overall goal of health research  capacity strengthening requires a bundle of interventions more than soft and methodological  capacities transfer: developing competitive  and structuring grant and fellowship schemes administered  by African institutions; improving research environment | Research institutions enhanced linkages with local representations  of international partners; By supporting south institutions in  developing their own protocols instead of merely implementing  proposals developed by international agencies or  North institutions, the NetSRH focuses not only  on the development of the methodological capacities but  also on the shift of power and responsibilities. | Unspecified |
| Elmusharaf, From local to global: a qualitative review of the multi-level impact of a multi-country health research capacity development partnership on maternal health in Sudan, 2016 | Sudan | Health systems research capacity development project for key decision and policy makers and academic staff in Sudan, part of the Connecting health Research in Africa and Ireland Consortium (ChRAIC1) | Unspecified. 1 part of the data collection came from 42 interviews  conducted by 14 locally trained women and 42  debriefing sessions conducted with these women; 13 critical  incident cases, and; 37 stakeholder interviews. | Case study | ChRAIC was an African/Irish health systems research  capacity strengthening consortium (2008–2015) established  with the aim of supporting the Irish Government’s  pro-poor development policy through conducting research  that would strengthen health systems in Africa  component 1: four year Ph.D programme on health systems research  component 2: lead institutions  were identified in each partner country and through  these institutions research teams were established on a  country-by-country basis. | National knowledge synthesis on  equity and access, governance and human resources in  health; identification of country level health systems  research priorities; research capacity assessment and  skills training | Graduation of a  Sudanese team member with a Ph.D.  Memorandum of Understanding at country  level between the Ministry of Health, research and  academic institutions in Sudan. The establishment of  country level initiatives and a research unit | The Ph.D. research has had an impact on the PEER  researchers and health promoters, the health service  officials and the community in which they work. The IPHE approach was recognized by international organizations (like Women Deliver) | Slow process of evolving the country team and  commitment to get people on board and involved from  an early stage enabled the development of long term relationships  and agreements that have continued beyond  the life of ChRAIC | Understanding of a partnership as a ‘network of action’ with the aim to achieve more than we can as individuals and to  have influence beyond the pilot site. | Delays in funding, access to documents and data |
| Ezenduka, Evaluating a capacity development intervention in health economics among producers and users of evidence in Nigeria: a case study in Getting Research Into Policy and Practice (GRIPP) in Anambra State, 2022 | Nigeria | Evaluate the impact of the workshop training  on selected stakeholders on the use of health economics evidence to inform health policy and practice in Anambra state. | 40 participants, comprising 12 producers and 28 users of  evidence from the SMOH, teaching hospitals and the  universities in the state. | case study using a pre-post test approach | A two-day capacity development workshop  was conducted to train the participants on the use of economic evidence to inform health policy and practice. | didactic and practical/group work sessions to .achieve the goals of developing health economics skills for informing efficiency and equity in decision  making and resource allocation, | The significant differences between the test scores indicated success in  increasing the knowledge of participants on the use of health economics evidence. | Results corroborated  participants’ perceptions that the workshop impacted positively on their ability to apply the knowledge of health  economic evidence to inform decision making in their respective practices. | the study provides useful insights on the capacity of relevant stakeholders to use scientific evidence  to inform policy and practice, in recognition of the poor  use of such evidence in LMICs for effective control of  diseases. | Findings underscore the need for significant  and regular upgrade of participants’ knowledge on the  use of health economic evidence to inform decision  making in the state’s healthcare system. | Small sample size. short period of training and assessment |
| Hamid, Learning Active Implementation Frameworks: the  role of implementation teams in a case study from  Pakistan, 2020 | Pakistan, Palestine’s West Bank, Egypt | Share the learning and outcomes resulting from the experience of the Maternal, Neonatal and  Child health (MNCH) Department of the Health Services  Academy (HSA) enrolled in the  Implementation for the Professional Learner–a multi-  country implementation practice of the Safe Childbirth Checklist (SCC) and science capacity-  building program developed and conducted by the  WHO Collaborating Centre at University of North  Carolina (UNC), United States. | 8 team members from MNCH department, HSA  alumni and clinicians. The implementation team was  composed of professionals of diverse background and experience, including public health professionals,  clinicians, sociologists and economists. | Case study | 9-module course spanning a period of 52 weeks delivered through a cloud-based Learning Management System (LMS) in the Obstetrics & Gynecology dept of a hospital. | Learning and coaching provided a virtual classroom with the facility to access reading materials, videos, presentations and recordings of the teaching sessions on implementation practice and research followed by a session where 3 teams presented their own project &  how the learning had supported putting into practice the innovations/interventions central to projects. | 4 stages of implementation project were reported including ‘exploration’ (assessing and creating readiness), ‘installation’  (amassing human and financial resources); and ‘initial  implementation’ activities and outcomes (supporting  the use of the innovation in practice) that eventually  lead to ‘full implementation’. | Outcomes included further enhancement of the  capacity of local health professionals in implementation science, systemic change and the  effective use of innovations in practice at sub-national/regional levels. | During the exploration, installation and initial  implementation stages identification of potential champions and best practices for the intervention supported  effective delivery of Implementation Team’s work. Selection of useable innovation by the  Implementation Team based on its merit, simplicity,  acceptability by the practitioners was important. | HSA can be trail blazer for devel-  oping such trainings and providing support to public  health professionals at sub-national, regional and  local levels. | Poor motiva-  tion of staff and resistance to change the status quo, in addition to poor or inaccessible  checklist supply. |
| Harpham, Bridging the gaps sector to sector and research to policy: linking family planning to urban development, 2021 | Urban Sub-Saharan Africa (SSA) | Build the policy-relevant evidence base on FP and fertility outcomes in urban areas of SSA, provide skill-building opportunities to early career researchers, engage with policy makers. | 16 young researchers, referred to as fellows, who completed graduate training in last ten yrs. | Qualitative, narrative testimonials | Training and mentoring well over 1-3 years as funds for policy outreach at local, national, and international levels. Virtual, networking via email, WhatsApp groups. | Trainings or review sessions (by the Africa Population & Health Research Center) on tools to improve policy engagement and communication and research-to-policy linkages | Feedback quotes | Program build multiple and multi-phasic mechanisms to maximize intersectoral and research-to-policy linkages. Researchers starting to probe the interactions of FP and urban-governance phenomena, plus interrogating some over-simplified dichotomies & preconceptions, enhanced | There’s enough knowledge and experience to train researchers how to engage and communicate with policy-makers but the leap from one sector to another is not strong if one-sided. | Unspecified | Limited outcomes reported, Difficulty in recruiting suitable fellows. Urban sector’s dynamic and complex nature was a particular challenge for FP researchers |
| Kabra, Research capacity strengthening for sexual  and reproductive health: a case study from  Latin America, 2017 | Paraguay | Identify the outcomes  and impact of the Human  Reproduction Program (HRP) efforts and to learn from these past efforts and contribute to the HRP’s future Research Capacity Strength-  ening (RCS)  programme by analyzing the 5 year period of research capacity strengthening at one institute in Paraguay. | 1 long-term institutional  development (LID) grant | Case study of 5-year history of LID | The LID  provided funding to the Paraguayan Center for Population Studies (CEPEP) for the implementation of well-defined research projects; research training, organization of short group learning activities; purchase of computer equipment and software, including establishment of information technology  facilities; data processing and library resources. A mentor- mentee relationship  developed between the two institutions with staff visiting each other’s institution to work together on developing and implementing research protocols. Reorganization  and expansion of CEPEP’s library. Support researchers  from LMICs to disseminate their research findings. | During the first 5 years of the LID grant (2009–2014), based on gaps identified by the  institution, training support was provided on data  analysis (quantitative/qualitative), multivariate analysis,  scientific writing, dissemination of research results, and  library management. | Following the training  on scientific writing and dissemination of research results,  there began a shift towards publishing in national and  international scientific peer reviewed–journals, developing  policy briefs, participating and organizing congresses and  seminars. Research results were published on intimate  partner violence, mental health, fertility decline, and  trends of caesarean delivery. Evaluation of the  National Reproductive Health Plan on RH of adolescents and young  women were published. A series of policy briefs produced to disseminate research findings to  authorities. Dissemination of findings wasn’t only limited to publications. | Other regions can similarly benefit from HRP’s strategy of re-  search capacity strengthening like Paraguay. Similar modalities and strategies of linking LID and RPM grants (within the region) be used in other  countries/regions and call for greater support from  development partners and governments for strength  ening research capacity to improve SRH. | Greater support is needed from developmental partners and governments to strengthen research  capacity in LMICs to improve SRH. Long term support to institutions to attain a certain  degree of capacity to conduct quality research in  sexual and reproductive health and to sustain the  gains made is critical. Increase collaboration between mature and  emerging research centres for further strengthening  the research environment. Networking and visibility of local institutions will help garner support, increasing recognition and  leading to sustainability. | The achievement of sustainability is evidenced in recent  years by the number of projects and seminars receiving  financial support from sources other than HRP. To advance research on SRH, researchers in LMICs  need continuous and long-term support to undertake  research based on national needs and priorities and to  disseminate research findings to SRH decision makers. | Unspecified |
| Keita, Strengthening equitable health systems in West Africa: The regional project on governance research for equity in health systems, 2022 | Nigeria, Sierra Leone, Burkina Faso and Senegal, | Describe a West African Health Organization (WAHO) supported an innovative regional initiative that contributes to building effective  decision making, community and researcher partnerships to strengthen equitable health systems and influence local programmes  and policies. | 56 project stakeholders, document review | qualitative, descriptive with key informant interviews | four research projects  targeting specific health system issues were  rigorously selected in four countries namely  strengthening of the health system through better,  equal access to primary healthcare (Nigeria);  barriers faced by pregnant women to free access to  health facilities (Sierra Leone); the development of a process for assessing the performance of the district health system (Burkina Faso); and funding, equity and governance in the health system  (Senegal). | designed an interview guide to collect information  on three main project sections; process, outcome  and impact.  review of published and unpublished (grey) materials  from national levels | A diverse range of stakeholders  perceived that the projects were in line with national priorities, were well managed and were equitably implemented. | The projects  generated evidence that could increase access to and improve quality maternal health services. | Study provides insight into project implementation in  West Africa, bearing in mind context-specific issues. | Sustainable partnerships were  formed and stakeholder and research team capacity were strengthened. | The documentation which did not cover the full  implementation period did not enable an in-depth  analysis to be made of the aspects linked with  knowledge transfer |
| Koso-Thomas, The Global Network for Women's and Children's Health Research: A  model of capacity-building research, 2015 | India, Pakistan,  Guatemala, Zambia, Kenya, the Democratic Republic of Congo, Argentina, Uruguay,  Tibet, Brazil | Demonstrate model for building local maternal and child health research capacity in resource-poor  settings | 102 research clusters in six countries | randomized clinical trials | Global Network grantees are  selected based on NIH review of meritorious applications which are  submitted in response to a request for applications | When a trial is approved for implementation  in the field, part of the preparation is identification of  research staff who will need to be trained in the various areas of  study implementation, enrollment and consent, data entry and  management | By virtue of their participation in the  Global Network, principal investigators, senior foreign investigators  and team members alike have received numerous academic  awards and recognition from their home institutions and the  health systems of their countries. Specifically, 26 Global Network affiliated investigators have completed Masters degrees, nine  PhDs or other doctorate degrees | Both the positive and negative  outcomes of the trials have been instrumental in driving the international  research agenda and health care practice in low  resource settings. |  | Developed a strong cadre of interdisciplinary health research teams  all  research staff participate in and receive accreditation in Good  Clinical Practice as well as additional research training and clinical  intervention training. | Issues related to  data reporting such as misclassification of cause of death. Though not formally studied,  contextual factors such as free health care provision, environmental  disasters such as floods, and sudden changes in personnel are  implicated in the data-reporting trends that are seen. |
| Millimouno, Evaluation of Three Blended Learning Courses to Strengthen Health Professionals' Capacity in Primary Health Care, Management of Sexual and Reproductive Health Services and Research Methods in Guinea, 2022 | Guinea | Evaluate the reasons for dropout and  abstention, the learners’ work behavior following the training, and the impact of the  behavior change on the achievements of learners’ organizations or services. | Out of 1,016 applicants, 543 including 137 (25%) women were enrolled in three courses. | cross-sectional  study using a mixed-methods approach was used. | Three blended courses on Primary Health Care (eSSP), Management of  Sexual and Reproductive Health Services (eSSR), and Research Methods (eMR) were  developed and implemented between 2017 and 2021 by the Maferinyah National Training  and Research Center in Rural Health, a training and research institution of the Ministry of  Health in Guinea. | The third course  on Research Methods (eMR) targeted medical students before  completing their thesis research component at the end of the  medical school and health professionals already or intending  to be involved in public health and research | Overall success rate (among all enrollees) ranged  from 50% (eSSP) to 58% (eSSR). The majority (87%) of the learners reported applying  the knowledge and skills they acquired during the courses through activities such as  supervision (22%), service delivery (20%), and training workshops (14%). | These findings showed fair success rates and a positive impact of the  training on learners’ work behavior and the achievements of their organizations. | A positive  impact of the training on utilization/coverage of services and increased revenues for their  health facilities were also reported by some trainees. | Unspecified | Participant dropout of 30%. Qualitative interviews were conducted  among a limited number of learners, and it relied on self-report  that is subject to bias. |
| Millimouno, Outcomes of blended learning for capacity  strengthening of health professionals in  Guinea, 2021 | Guinea | Analyze the outcomes of blended learning for post-Ebola capacity strengthening of health professionals in Guinea. | The quantitative strand included 282 doctors,  nurses, midwives, community health workers, public  health technicians, and last-year medical school students. The qualitative strand focused on 8 learners. | Cross-sectional study using a mixed-methods approach | Two courses lasting 3 months each (7–8 modules) were developed and implemented: one in Primary  Health Care (eSSP) and the other in Sexual and Reproductive Health Services Management (eSSR). | Both eSSP & eSSR courses were offered online on the Moodle platform, followed by a face-to-face capacity-building workshop. | According to learners’ feedback, the blended learning activity  enabled capacity building, particularly in monitoring a  health area and data management. Most learners strongly agreed that the courses are relevant for targeted health professionals. | Two blended courses for capacity strengthening of health professionals were successfully developed  and implemented in Guinea. | Developing the modules consumes time and requires  sufficient human resources to develop the contents, implement the course according to the model used, plan  prompt and constant mentoring.  Face-to-face sessions are essential for building learners’ capacity and obtaining their opinions to improve the quality of the course. | Built local  institutional capacity (Maferinyah e-learning team) in  designing blended courses, developing modules, and  independently managing the learning platform. | Didn’t assess impact of the training on  learners’ everyday professional activities.  Variables entered into  regression analysis have a narrow scope. Qualitative interviews had limited scope. |
| Pinto, International participatory research framework:  triangulating procedures to build health research  capacity in Brazil, 2011 | Brazil | Use International Participatory Research Framework (IPRF) to build a partnership to study the roles of com-  munity health workers (CHWs) in Brazil’s Family  Health Program (PSF) focused on HIV prevention | Nearly 200 CHWs | Mixed methods, case illustration | Training partnership between Brazil & US based researchers and CHWs | The pre-research period lasted 1 year and the research itself took 3 years to be completed. | By using the IPRF, the partnership  achieved several participatory outcomes: community-  defined research aims, capacity for future research and  creation of new policies and programs. They engaged  CHWs who requested that they study their training needs,  and they engaged CHWs’ supervisors who used the data  collected to modify CHW training. | Researchers and community partners  can use the IPRF to build partnerships in different  international contexts. | By triangulating steps and actions,  the IPRF advances knowledge about the use of community-based participatory  research  methods/procedures for international health research. | Data collected from  CHWs will form the basis for a grant to test CHW  training curricula. | The IPRF has been implemented in a few different contexts, but has not been fully evaluated  in terms of partnership longevity. Evalua-  tions of the IPRF will be needed to appraise  how useful it is for maintaining partnership over time, sustainability of community changes. |
| Ropa, Lessons from the first 6 years of an  intervention-based  field epidemiology  training programme in Papua New  Guinea, 2013–2018, 2019 | Papua New  Guinea | strengthen  the public health workforce and health  systems across all levels of government | The program consists of five training phases  over 8 months. Each cohort consists of 15–18  health workers (Fellows) supported by a team of trainers  and mentors (faculty).  Fellows from provincial and district health offices  prioritized. | Case study | Papua New Guinea’  National Department of Health and its partners developed  a field epidemiology training programme of Papua New  Guinea (FETPNG) to strengthen the country’s public health  workforce. The training programme covers field epidemiology  competencies and includes the design, implementation and  evaluation of evidence-based  interventions by Fellows. | curriculum is focused on developing competencies  that are retained, applied and lead to measurable  impacts | graduated  81 field epidemiologists who completed projects | At  least one FETPNG Fellow has graduated from every PNG  province, as well as the Autonomous Region of Bougainville  and the National Capital District. This target was met  in 2016, 1 year ahead of the 5-year  target.  The most common focus area for intervention projects  was tuberculosis | Administering the program with in the Ministry of Health allowed for more flexibility than going through an academic institution | Retention of graduates within the government  system is a key outcome that supports the long-term  objectives  of strengthening health systems and developing a  network of response-ready  public health professionals  across the country.  As research topics vary additional faculty areas of expertise will be needed | Mixed-methods  evaluation  underway to understand the impact of FETPNG  training as well as barriers and enablers to translating knowledge into action. |
| Serruya, EviSIP: using evidence to change practice through  mentorship – an innovative experience for  reproductive health in the Latin American and  Caribbean regions, 2020 | 16 countries in Latin America & Caribbean | Detail  the concept, processes, methods, procedures and  results of EviSIP [Evidence from Perinatal Information System (SIP)] to support future  mentorship, especially among people and institutions  from LMICs in the analysis  of local healthcare information, to improve sexual  and reproductive healthcare in women. | Total number unclear, Junior researchers/clinicians from  these countries were included, along with expert researchers in RH from  across the world. Small grant proposal were awarded | Case study describing implementation of this  initiative in reproductive healthcare facilities using the SIP. | 4-day EviSIP meeting, 6 wk distance mentorship process,  followed by a 4-day face-to-face immersion in  mentoring, as well as intensive data analysis and  writing. Selected healthcare professionals participated in  one-hour once-weekly online meetings, where they received new information on research methods related to different areas of interest. Meetings included information on  how to write a scientific manuscript, review bibliography and other research priorities. Optional research  training with a two-month online course offered by  the Latin American WHO-HRP hub. | Representatives of selected centers from 10  countries shared their databases on maternal near  miss and/or abortion and addressed their relevant questions in the meeting. Participants learned to use information stored on the database and plan, present and analyze their data to produce a manuscript with  valuable knowledge. | By the end of the EviSIP meeting, all  groups had submitted a manuscript draft. 6 manuscripts sent to a peer-reviewed journal, 11 in final editing and 5 still in the writing phase. | The EviSIP exercise showed that the  teams need to work on technical capacity building, to  improve their work and data collection in a strategic  and scientific manner. The data collected can be used  as local evidence, in addition to input to feed national  and regional health-related informed decision-  making and improve the conditions, healthcare and services provided. | Networks  allowed local and regional clinicians to expand their  knowledge and skillset in the field of research. As  a result, this process not only built local capacity,  especially in post-abortion and postpartum care but also provided an opportunity to improve surveil-  lance and create and impact on quality of care. | The staff and some international experts participating in the initiative are still committed to sup-  porting those researchers so that their  manuscript can be accepted for publication. | Time was restricted to write & submit projects. Participants had a diverse  background and experience. Participants had limited time to work with mentors before their face-to-  face meeting. |
| Sow, Strengthening local capacity for abortion-related research in contexts with highly restrictive abortion laws: The case of STARS in Mali, 2022 | Mali | strengthen local capacity for abortion research | 50 researchers from the University of Bamako, medical researchers,  university professors and public leaders in Mali | multifocal competency-  based pre-and post-program intervention | several training sessions in critical research  skills including qualitative and quantitative  interviewing, and use of qualitative and quantitative  analytical software and referencing applications.  Software was procured for research team members.  STARS has also held workshops on research ethics,  protocol development, sampling, tool development,  and research report writing. | Experiential  learning and hands-on training support. online  database of research, tools, training materials and  other resources related to abortion. | the program is evolving  satisfactorily, Various key issues related to abortion in Mali remain  uninvestigated, and studies on these could provide  additional capacity strengthening opportunities for more researchers. | The program’s learning-by-doing approach has boosted the skills of individual researchers while also enhancing institution- based abortion and SRHR research expertise in Mali. | Restrictive abortion  laws do not translate into a lack of interest in research. Programs interested in  building capacity for research on sensitive issues must be bold in disregarding  assumptions and in focusing learning locally. | Ultimate results will depend on the sustained commitment of funders to the program in the  full realization that capacity building requires long-term investment and support for it to fully bear fruits. | Widespread cultural and religious conservatism continues to be a challenge. Movement restrictions and shelter-  in-place directives during COVID. |
| Srisaeng, Looking toward 2030: Strengthening midwifery education  through regional partnerships, 2019 | Thailand, Laos | Describe the impact of a regional capacity‐building project between Thailand and Laos that supports the UN’s SDG 3 through midwifery education. | 9 health science schools in Laos, the trilateral South‐South Cooperation (SSTC) work plan be‐  tween Laos Ministry of Health, Thailand International Cooperation  Agency and United Nations Population Fund (UNFPA) was initiated. | Discussion paper based on exemplar | 2‐year project (October 2015–  November 2017). Phase 1 needs assessment used in-depth interviews focused on knowledge of didactic teach‐  ing, clinical supervision, and skills in curriculum organization with  the goal of identifying the current state of midwifery education  and competencies of midwifery educators. | In Phase 2, Two groups of 28 and 24 participants enrolled in 2 sections of training program titled, “Strengthening Midwifery Educators in  Lao PDR”, respectively over 6 months. Domain 8 focused on research. Follow‐up evaluation was initiated 4 months after the training to assess application of knowledge and clinical skills into their work as  midwifery teachers and preceptors. | Knowledge gained  increased the teaching confidence of midwifery educators while  linking international standards and competencies to curriculum revision. | Follow‐up evaluation visit to those who completed the course demonstrated that the  newly trained midwife educators improved their knowledge, skills,  and attitude and were able to make effective changes to their teach‐  ing. As a result, training was expanded to a third group in 2018, so more educators would be trained to achieve International Confederation of Midwifery (ICM) standards  and WHO core competencies. | Partnerships especially those between countries in the same region, are crucial to the  success of meeting the SDGs. | Regional partnerships may be highly effective in creating sustainable capacity‐building projects. | Due to time and budget limi‐  tations, only two schools were purposely selected for evaluation. |
| Tomatis, Evidence-based medicine training in a resource-poor  country, the importance of leveraging personal and  institutional relationship, 2011 | Perú | Describe the results of an international  effort to improve research capacity in a developing country sponsored by programs for AIDS and TB control. | 220 clinicians | Quasi-experimental (before–after), assessed self-reported competence and importance in EBM using a Likert scale (1 = low, 5 = high). | Interactive lectures and case-based workshops. | Over 4 yrs, an annual 3-day course in Perú | For phase I, self-reported EBM  competence increased from a median of 2 to 3 and the perceived importance of  EBM did not change. For phase II, before the course, 8–72%  graded their competence very low. After the course, 67–92% of subjects  graded their increase in knowledge very high. | The course had measurable improve-  ments of self-reported competence, perceived utility and readiness to incorporate EBM for HIV/AIDS and TB into  their practices. | Translational research and building the  research capacity in developing countries is critical for translating best available evidence  into practice. | The  partnerships developed during this project have resulted in broader  training plans including formal collaboration agreements between  the participating schools. | Limited availability of studies relevant to the local reality written in Spanish, participants’ limited time and lack of long-term follow-up on practice change. |
| Tran, Developing Institutional Capacity for Reproductive Health in Humanitarian Settings: A Descriptive Study, 2015 | 49 LMICs, 33 HIC | Gain insight into the overall state of reproductive health in humanitarian settings (RHHS) over the past  decade from an institutional perspective, by describing the capacity of government, non-government, United Nations, humanitarian, and development institutions to address RHHS. | Respondents represented 82 institutions from 48 countries, of which two-thirds originated  from LMICs. | Descriptive, Online questionnare administered in English & French with open and closed questions. | The purpose of the questionnaire was to capture data about and gain insight into institutional  capacity for RHHS along the emergency to development continuum including DRR, crisis  response, early recovery and redevelopment, with trends in organizational changes over time. | none | Institutional areas of work included capacity development, program delivery, advocacy/policy work, followed by research and donor activities. Except for abortion-related services, respondents cited improved efforts in advocacy, capacity develop-  ment and technical support in their institutions for RHHS to address clinical services,  including maternal and newborn health, sexual violence prevention and response, HIV pre-  vention, management of sexually-transmitted infections, adolescent RH, and FP. | Overall growth in institutional capacity in RHHS over the past decade,  indicating that the field has matured and expanded from crisis response to include RHHS into elements of the emergency management cycle. | It is critical to consoli-  date the progress to date, address gaps, and sustain momentum. | The positive development of institutional capacity globally is also suggested by the overall  trend in organization expenditure. | Results need to be carefully interpreted as  they only allow us to glean some insight into self-reported organizational capacity, which does  not reflect quality of or access to services on the ground. |
| Tulloch, Using research to influence sexual and  reproductive health practice and implementation  in Sub-Saharan Africa: a case-study analysis, 2011 | Ghana, South Africa, Tanzania | Give voice to researchers based  in Southern institutions and to explore SRH research to  policy and practice interactions in Sub-Saharan Africa. | Findings from SRH/HIV research programs in SSA. | Case study analysis | Case-studies presented at a meeting  on research engagement with policy and practice in SRH and HIV, which involved researchers, communication specialists and donors working with DFID-funded  SRH and HIV Research Programme Consortia. | none | The analysis emphasizes the relationships and communications involved in using research to influence  policy and practice and recognises a distinction whereby practice is not necessarily influenced as a result of policy  change – especially in SRH – where there are complex interactions between policy actors. | There’s a real need  to think about the practical implementation of a policy  from the inception of a program; we need to move  beyond conceptualising a linear process that begins with  conceptual use of research evidence (tackling attitudes  and knowledge) and finishes with instrumental and  attributable use. | The case-studies illustrate the  importance of long-term engagement between researchers and policy makers and how to use evidence to  develop policies which are sensitive to context: political, cultural and practical. | Researchers and communication  specialists need to work at developing networks of  actors across the policy and practice continuum, while  designing long-term communications strategies appropriate to a range of specific technical, political and cultural contexts. This | Unspecified |
| Vaz, Enhancing the Education and Understanding of Research  in Community Health Workers in an Intervention Field  Site in South India, 2014 | India | Describe a Professional Development Program  (PDP) to optimize the quality of the research, and to make involvement in research  more meaningful for all members of the research team on HIV, TB, malaria. | Total number unclear | Mixed methods, pre-post test, focus group discussions (FGDs) (5-10 per group) | Training modules as interactive 2 to 3 hour sessions  with PowerPoint presentations, interspersed with  activities to enhance research understanding and application. Basic and advanced modules presented. | Pre and post-tests were  conducted for all modules, qualitative assessment after 3 yrs. Individuals who didn’t score at least 75% on the post-test had sessions  in smaller groups or individually until they passed. | While initial 'pre-test' knowledge across modules was lower amongst community level field  workers than staff of higher cadres, they achieved passing grade scores (>75%) with the program.  Longer term retention of knowledge over 6 months for the basic modules, was in excess of 75% for all  staff. | FGDs revealed that while many in the research team began work with an  incomplete understanding of research methods and community health principles, investing time and resources in education beyond protocol training via the program realized long-term benefits to the PDP study and individual staff, particularly community level workers. | The program had a positive learning outcome and  return on the money and time invested. There is a need to enhance the capabilities of CHWs to be part of  a research team in field settings, to optimize their performance while utilizing their existing  skill sets. | In the long term, a certification of CHWs would allow community level  workers the opportunity to work in the broad area of  community based research with recognition and  dignity. | A few participants felt that the  sessions were too long, and that there was some  variability in the amount of interaction by module. |
