# Supplementary material for: Sexual and reproductive health research capacity strengthening programs in low- and middle-income countries: A scoping review
Source: PLOS Glob Public Health. 2024 Oct 3;4(10):e0003789. doi: 10.1371/journal.pgph.0003789 (PMC11449353; doi:10.1371/journal.pgph.0003789)
Supplement: Attachment — Submitted filename: Updated PLOS RCS Response to Reviewers.docx [file pgph.0003789.s003.docx]

October 24, 2023

Dr. Catherine Kyobutungi

Professor Madhukar Pai

Editors-in-Chief

*PLOS Global Public Health*

San Francisco, California, US

**Title: "Reproductive health research capacity strengthening programs in low- and middle-income countries: A scoping review” (PGPH-D-23-02103)**

Dear Drs. Kyobutungi and Pai:

We are pleased to submit our article titled “Reproductive health research strengthening programs in low- and middle-income countries: A scoping review” to be considered for publication in *PLOS Global Public Health*. We revised our original submission, PGPH-D-23-01513, to respond to the concerns that the literature search was completed over a year ago.

**Editorial comments:** In the case of your submission, we are concerned that the literature review was completed in November 2021. Systematic and scoping reviews should provide an up-to-date overview of the research topic covered. Since the literature search was completed over a year ago, we are concerned that this study does not meet this requirement. As a result of these concerns, we cannot consider the manuscript for publication at PLOS GPH at this time.

However, if you are able to address the concerns discussed above, we may be able to consider a revised version of the manuscript. This would however require a substantial amount of work and thus, would be considered as a new submission. If you are able to revise the manuscript to respond to the concerns noted above, please refer to the original submission in the cover letter and accompany the submission by a rebuttal letter that responds to the comments made on the original manuscript.

**Response to editorial comments:** Recognizing that scoping reviews should provide an up-to-date overview of the research topic covered, we updated the literature search in August 2023 and incorporated more recent results into the text. We referred to the original submission in the cover letter.

Sincerely,

The Authors
