# Supplementary material for: Sexual and reproductive health research capacity strengthening programs in low- and middle-income countries: A scoping review
Source: PLOS Glob Public Health. 2024 Oct 3;4(10):e0003789. doi: 10.1371/journal.pgph.0003789 (PMC11449353; doi:10.1371/journal.pgph.0003789)
Supplement: Attachment — Submitted filename: Revised Updated RCS Scoping Review Response to Reviewers.docx [file pgph.0003789.s004.docx]

19 March 2024

María De Jesús Medina Arellano, PhD

Academic Editor

PLOS Global Public Health

**Title: “Reproductive health research capacity strengthening programs in low- and middle-income countries: A scoping review” (PGPH-D-23-02103)**

Dear Dr. María De Jesús Medina Arellano, Academic Editor, PLOS Global Public Health:

We are very pleased to have the opportunity to revise our original manuscript for publication in *PLOS Global Public Health*. We addressed each of the reviewer’s comments and made the necessary revisions and modifications. Responses are provided below.

**Reviewer #1**

**Comment #1:** It is very common to treat the “sexual and reproductive” health or rights, when reproductive aspects are analyzed. Gender identity, gender expression, sexual orientation, etc. are no necessarily regularly considered, and their impact in health are very important. I think it is important to reconsider that in the tittle is written “Reproductive health research capacity”, a key word is “Reproductive health”, and in the introduction there is an ampliation to “Sexual and reproductive health”. It should be uniform.

**Response #1:** Thank you for this suggestion for improvement. To improve uniformity, we updated the title to include “Sexual and reproductive health”.

**Comment #2:** There is a relatively old discussion regarding the relationship between research an public policies. The introduction says “There remains a limited understanding of the effectiveness of SRH research strengthening programs to raise skill sets, publications, and infrastructure and ultimately influence health policy and patient outcomes in LMICs.” Health research has its own logic, but also policies have another logic. Maybe, in an ideal world, policies should be guided by evidence (Ham C, Hunter DJ, Robinson R. Evidence based policymaking. BMJ. 1995 Jan 14;310(6972):71-2. doi: 10.1136/bmj.310.6972.71.) However, this has been considered at least as an incomplete vision of the problem (Azuonye IO. Evidence based policymaking. Academic experts don't have a monopoly on wisdom. BMJ. 1995 Apr 29;310(6987):1141. doi: 10.1136/bmj.310.6987.1141b.)

**Response #2**: Thank you for highlighting the complexity of how health policy is formulated and that health researchers are not poised to offer complete policy solutions, per se. We incorporated an acknowledgment of this complexity in our text by softening our language. We changed the text to read “Strengthening our understanding in this realm will help to inform health policy in LMICs” thus acknowledging that health research is one important force shaping policy, but not the only one.

**Comment #3:** In the field of reproductive health (or sexual and reproductive health) this is known that “Sexual and reproductive health and rights (SRHR) are fundamental to people's health and survival, to economic development, and to the wellbeing of humanity.” (Starrs AM, et al. Accelerate progress-sexual and reproductive health and rights for all: report of the Guttmacher-Lancet Commission. Lancet. 2018 Jun 30;391(10140):2642-2692. doi: 10.1016/S0140-6736(18)30293-9.) Legal framework and public policies are as relevant as research in reproductive health.

**Response #3:** We appreciate this observation and updated the text to make the point that legal framework and public policies are as relevant as research in reproductive health. We cited the above mentioned article in the Discussion, page 20.

**Comment #4:** 4th. A clear example about previous topic is abortion. Recent research on abortion classification shows that “When grouped by the legal status of abortion, the proportion of unsafe abortions was significantly higher in countries with highly restrictive abortion laws than in those with less restrictive laws.” (Ganatra B, et al. Global, regional, and subregional classification of abortions by safety, 2010-14: estimates from a Bayesian hierarchical model. Lancet. 2017 Nov 25;390(10110):2372-2381. doi: 10.1016/S0140-6736(17)31794-4.) It is common that LMICs have restrictive abortion laws as well restrictive access to reproductive rights (or sexual and reproductive rights).

**Response #4:**  We incorporated the example of abortion into a paragraph inserted into the Discussion section about the importance of legal frameworks and public policies. We also cited the Ganatra article in the Discussion, page 20.

**Comment #5:** Normative aspects should include not only legal ones, but ethical too. Authors say that “when participating in the research, they are often not given appropriate credit”. They could cite that “Guideline 8: Collaborative partnership and capacity-building for research and research review” (International Ethical Guidelines for Health-related Research Involving Humans (https://cioms.ch/wp-content/uploads/2017/01/WEB-CIOMS-EthicalGuidelines.pdf) which analyzes the problem and propose some solutions.

**Response #5:** We expanded the Introduction, lines 98-94, to cite some solutions proposed in reference recommended above, “Guideline 8: Collaborative partnership and capacity-building for research and research review”.

**Reviewer #2**

**Comment #1:** This is a potentially useful review.

**Response #1:** Thank you!

**Comment #2:** The authors state that there is limited understanding of the effectiveness of SRH research strengthening programs to raise skill sets, publications, and infrastructure and ultimately influence health policy and patient outcomes in LMICs and that more information is needed to understand how SRH research is sustained after program completion. It is not clear how these stated gaps in literature are factored into the study. Is the goal of the review to describe literature on the effectiveness of SRH RCS or to describe the the format, duration, and topics covered of program interventions? A reader gets the feeling of a disconnect between the stated gaps and the study findings. The authors could focus merely on describing the interventions and the outcomes as described in the published literature. The study does not resolve/ answer the mentioned question of the effectiveness of RCS intervention. This means the paper has to be reintroduced to focus on the issues addressed in the review.

Answering the question of effectiveness would require a review of assessments/evaluation of SRH RCS focused-work.

**Response #2:** The reviewer is correct that we are focusing on describing the interventions and the outcomes as described in the published literature, rather than if the effectiveness is sustained after the intervention. As requested, the introduction was adjusted to reflect this.

**Comment #3:** It will be great if the authors could clarify how they dealt with papers that focused on RCS in the field of public health generally. Some published studies on RCS interventions in public health include trainees who work on a diversity of health related topics of which SRH will be one. Did such paper qualify for inclusion? My point is that some RCS program include SRH but as a component of health generally.

Further questions include: how do such programs ensure gender diversity? Do they have built-in tracker systems; what are they key research skills focused on and not focused on etc.

The authors' point that "More energy must be directed toward correcting power imbalances in capacity strengthening initiatives" appears casually thrown in and does not reflect anything from the review.

Rethinking the paper with my first and second point above in mind will help improve the value of this manuscript.

**Response #3:** Studies that included the training of public health providers were included if they met all other inclusion criteria even if the entire program was not focused on SRH. Our key search words were: (1) sexual and reproductive health, (2) research strengthening or capacity building, and (3) low- and middle-income countries (LMIC) or low-resourced regions.

We updated the text to reflect that the articles did not address gender diversity and did not mention built-in tracker systems. We also inserted a paragraph into the Results section, pages 14-15 summarizing the research skills focused on in the RCS programs.

We appreciate the suggestion for improvement and removed the point that “More energy must be directed toward correcting power imbalances in capacity strengthening initiatives” since it does not reflect anything from the review.

We thank you for your consideration of this manuscript for publication and look forward to hearing from you.

Sincerely, The Authors
