# Supplementary material for: Sexual and reproductive health research capacity strengthening programs in low- and middle-income countries: A scoping review
Source: PLOS Glob Public Health. 2024 Oct 3;4(10):e0003789. doi: 10.1371/journal.pgph.0003789 (PMC11449353; doi:10.1371/journal.pgph.0003789)
Supplement: Attachment — Submitted filename: New RCS Scoping Review Response to Reviewers.docx [file pgph.0003789.s005.docx]

12 July 2024

María De Jesús Medina Arellano, PhD

Academic Editor

PLOS Global Public Health

**Title: “Reproductive health research capacity strengthening programs in low- and middle-income countries: A scoping review” (PGPH-D-23-02103)**

Dear Dr. María De Jesús Medina Arellano, Academic Editor, PLOS Global Public Health:

We are very pleased to have the opportunity to revise our manuscript for publication in *PLOS Global Public Health*. We addressed each of the reviewer’s comments and made the necessary revisions and modifications. Responses are provided below.

**Reviewer #1**

**Comment #1:** I consider the requirements were fully fulfill.

**Response #1:** Thank you!

**Reviewer #3**

**Comment #1:** The authors were responsive to the previous reviews, and I just have a couple of minor suggestions to further improve the manuscript. First, I noticed that the revision from "reproductive health" to "sexual and reproductive health" was in response to previous reviews, and I agree with the revision. However, the introduction still seems focused on reproductive health or RH, and it gets a little confusing. Please double-check for consistent terminology throughout.

**Response #1:** Thank you for recognizing our responsiveness to previous reviews, including the revision from "reproductive health" to "sexual and reproductive health". In the Introduction and throughout the manuscript, we double-checked for consistent terminology and updated the text.

**Comment #2:** It would be helpful to explain and justify why the timeframe of 2011-2023 was chosen.

**Response #2**: We appreciate this suggestion for improvement and added text at the end of the first paragraph in the Materials and Methods section to explain and justify why the timeframe of 2011-2023 was chosen.

Our revised text states: “The timeframe of 2011-2023 was chosen to capture the most recent and relevant literature in the field. This period ensured coverage of current research trends, methodologies, and findings while managing the scope of the review. Focusing on the last decade also aligns with the need to explore contemporary issues and developments in SRH research strengthening programs in LMICs.”

We thank you for your consideration of this manuscript for publication and look forward to hearing from you.

Sincerely, The Authors
